# Viruses' Life History: Towards a Mechanistic Basis of a Trade-Off between Survival and Reproduction among Phages

**DOI:** 10.1371/journal.pbio.0040193

**Published:** 2006-06-13

**Authors:** Marianne De Paepe, François Taddei

**Affiliations:** **1**Laboratoire de Genetique Moleculaire, Evolutive et Medicale, University of Paris 5, INSERM, Paris, France; University of Newcastle upon TyneUnited Kingdom

## Abstract

Life history theory accounts for variations in many traits involved in the reproduction and survival of living organisms, by determining the constraints leading to trade-offs among these different traits. The main life history traits of phages—viruses that infect bacteria—are the multiplication rate in the host, the survivorship of virions in the external environment, and their mode of transmission. By comparing life history traits of 16 phages infecting the bacteria *Escherichia coli,* we show that their mortality rate is constant with time and negatively correlated to their multiplication rate in the bacterial host. Even though these viruses do not age, this result is in line with the trade-off between survival and reproduction previously observed in numerous aging organisms. Furthermore, a multiple regression shows that the combined effects of two physical parameters, namely, the capsid thickness and the density of the packaged genome, account for 82% of the variation in the mortality rate. The correlations between life history traits and physical characteristics of virions may provide a mechanistic explanation of this trade-off. The fact that this trade-off is present in this very simple biological situation suggests that it might be a fundamental property of evolving entities produced under constraints. Moreover, such a positive correlation between mortality and multiplication reveals an underexplored trade-off in host–parasite interactions.

## Abstract 2

A comparison of life-history traits of 16 phages infecting *E. coli* reveals that although these viruses don't age, there is a trade-off between mortality and growth rate, which parallels that observed in many other species.

## Introduction

A fundamental assumption underlying many theories on the evolution of life history traits is the existence of constraints, leading to trade-offs, between various aspects of the life cycle [ 1, 2]. For higher organisms, such as mammals, fishes, and birds, a major trade-off has been observed between fertility and longevity [ 3]. The mechanisms that are most commonly proposed to explain such a relation are the existence of metabolic constraints and the allocation of finite resources either to reproduction or to survival [ 4, 5]. Based on this assumption, as an individual's fitness improves with an increase in its reproductive capacity, but is diminished by mortality; highest fitness is thus achieved as a compromise between these two traits. Theoretically, this should be true for any evolving entity that maximizes its fitness under constraints of limiting resources. These resources may be those produced by the organism itself, but they could also be someone else's resources as in the case of parasites that exploit their hosts.

To our knowledge, the existence of a trade-off between parasites' multiplication rate and survival has not been properly investigated. In the case of parasites, other relationships between traits are well known, such as the one between rapid exploitation of the host and the duration of the infection [ 6], or the impact on their virulence of the survival of parasites between hosts [ 7]. Over an important period of their life cycles, parasites transmitted via the environment have to survive outside their host before meeting a new susceptible host. Parasite survival in the environment is thus of great importance for an effective transmission. This is true of model organisms such as coliphages, which are phages that infect the bacteria Escherichia coli. Such phages have to survive between the bacteria they have just left and the one they will infect next, and there may be considerable variations in the period of time between these two events. Indeed, after reproduction on susceptible and accessible E. coli cells in an animal gut [ 8, 9], phage particles are excreted in the external environment. In rare cases only, they can be transmitted to a new gut where they may meet new host bacteria. Therefore, phages might have then to survive between hosts for an extended period of time. Hence, if we consider that viruses are evolving entities, one can test the validity of the predictions of life history theory by examining whether the trade-off between survival and reproduction also applies to viruses, even though they do not have a metabolism of their own.

Little information is available on the determinants of the stability of phage particles. However, as some E. coli phages are model organisms for molecular and structural biology, complete structural information is available and allows investigation of the physical forces involved in the inactivation of viral particles or virions. Virions consist of an RNA or DNA genome packaged into a proteic capsid, and some virions also include lipids or a proteic tail. A strong pressure is exerted on the inside of the capsid by strong repulsions between the highly charged neighboring nucleic acid segments, as well as by forces caused by the bending of the DNA [ 10, 11]. Experimental [ 12] as well as theoretical [ 10] studies have established that the forces inside the capsid reach values on the order of 50 piconewtons, which corresponds to 50 times standard atmospheric pressure.

Several studies have proposed that the major lethal event in harsh conditions, i.e., under heat or osmotic shock, is the release of the nucleic acid from phage particles [ 13– 15]. Indeed, deletion mutants proved to be more resistant, particularly to heat shock [ 13, 14, 16]. Conversely, virions of phages with genomes larger than the wild type are highly unstable [ 17]. It was concluded from these observations that genomic deletions decreased the internal force exerted by the packaged genome on the capsid, and therefore enhanced virion stability. These results were extended by studies showing that heat-resistant mutants are also more resistant to osmotic shock [ 14], extreme pH levels, high salt concentrations, and chemical treatments such as chlorination [ 18]. Direct observations of phage particles by electron microscopy have shown that the proportion of damaged phage particles increases proportionally with the loss of infectivity of phage populations [ 15], supporting the hypothesis that virion inactivation in stressful conditions is due to the rupture of the capsid.

The multiplication rate in the bacterial host, which is another essential aspect of the life cycle of these parasites, has been measured for almost every newly isolated virus, using classical techniques [ 19]. After infection of a host in an appropriate physiological state, new phage particles are synthesized, leading, in general, to the rapid lysis of the bacterial cell and the concomitant release of the newly formed virions. The multiplication rate of phages can be determined by two parameters: the burst size (the number of particles released in one cycle of infection) and the latency period (the time between infection and lysis of the host). Even among phages infecting E. coli cells in similar growth conditions, large differences can be measured in burst sizes and latency periods [ 20].

Here, we determined the life history traits of coliphages by measuring the decay rate and the multiplication rate of 16 well-characterized phages of E. coli—λ, M13, MS2, Mu, P1, P2, P4, ϕ80, ϕX174, PRD1, R17, T2, T4, T3, T5, and T7—under identical laboratory conditions. These phages were selected as being the most well-known representatives of each important family of phages [ 21], or the less well-studied close relatives of these phages: T2 (relative of T4), T3 (relative of T7), R17 (relative of MS2), and ϕ80 (relative of λ). With the exception of these pairs of related phages, the other studied phages cannot be placed within a phylogenetic relationship [ 21], ensuring that the effects reported cannot be due to phylogenetic constraints. Decay rates proved to be constant with time for all phages, indicating an absence of aging, but are very variable between phages. To understand this variability, we made an analysis of covariance between the decay rate and other traits of phages, such as structural properties of the virions and life cycle characteristics. This analysis revealed that the decay rate is positively correlated with the multiplication rate in the host, suggesting that phages have evolved a continuum of different reproductive strategies constrained by a trade-off. Moreover, two physical characteristics of virions, the density of the packaged genome and the thickness of the capsid, are correlated with mortality.

## Results

### Characteristics of Phage Decay

The kinetics of mortality of a population not only gives information about how fast death occurs, but by describing the distribution of the age at death throughout the population, it gives information about the processes that lead to death. We determined the precise kinetics of inactivation of the 16 phages previously listed, from a population of approximately 10 ^8^ viable phages down to 10 ^2^, by measuring the number of phage particles that are able to complete an infectious cycle when plated on the appropriate host, at different time points and at different incubating temperatures. Phage stability is very dependent on salt concentration and osmotic pressure [ 19]; so we used the standard high-salt LB broth instead of any defined medium that could favor one phage over another. For every phage, and at each temperature, the number of viable particles decreases exponentially with time ( Figure 1A). This indicates that these phages do not age; i.e., their probability of dying does not increase with time, which is probably linked to their absence of metabolism, repair, and redundancy. Indeed, the exponential decay of these phages is representative of a single-step process [ 22]. If two or more successive events were necessary to cause inactivation, we would observe an increase in the probability of dying since the number of viable particles that can be affected by the lethal event would increase. Although some changes in capsid characteristics may occur over a period of time prior to inactivation, these changes do not affect the ability of the phages to infect and multiply under the conditions we used.

**Figure 1:**
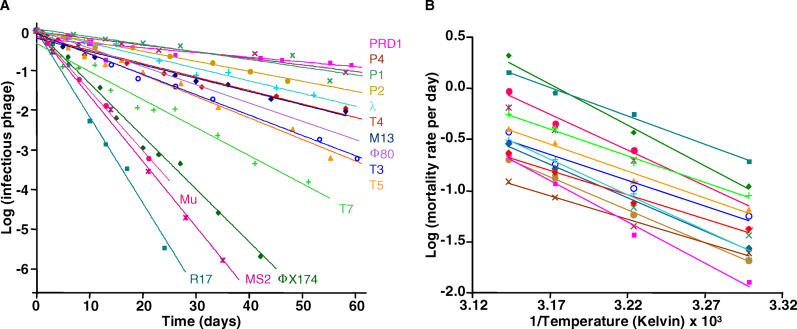
Mortality Rates of Phage Particles (A) Representative survival curves of phage particles maintained in LB at 37 °C, in the absence of host cells. Phage stocks are obtained by infecting growing E. coli host culture followed by cell elimination. Lines show exponential regressions, with *R ^2^* values ranging from 0.87 for P2 to 0.99 for MS2. The mortality rate is not influenced by the initial concentration of the phage populations (unpublished data). Each experiment was repeated at least three times independently. (B) Relation between mortality rate and temperature. Symbols are the same as in (A). Lines show exponential fits between the mortality rate and 1/T. *R ^2^* values range from 0.937 for Mu to 0.999 for P2.

To account for the possibility of phage interactions with free host cell receptors in the incubation media, we have shown with very different phages, T4, λ, and ϕX174, that most of the inactivation is independent of the initial concentration of phage in the incubation medium (unpublished data). Moreover, we observed populations of phages T4, T3, and λ by electron microscopy after incubation at 37 °C. We show that for the three phages, the proportion of broken virions increases after 8 d in the same proportion as the loss of ability to infect a susceptible host ( Figure 2). This result is in line with those published in similar conditions for phages λ and ϕ80 [ 15].

**Figure 2:**
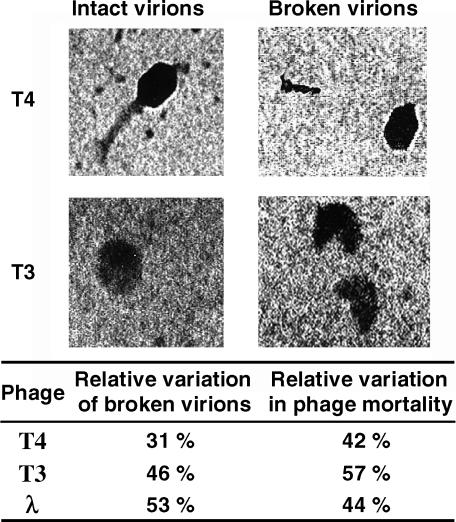
Observation of Virions by Electron Microscopy after 8 D of Incubation at 37 °C The table gives the relative variation in the proportion of broken virions, as measured by electron microscopy, of more than a hundred particles, as well as the variation in the number of viable phages measured by plating on a susceptible host. Both observations have been made on the same samples. For each type of phage, the proportion of broken virions is significantly higher after incubation, and the variation in broken-virion particles measured by electron microscopy is similar to the variation in the number of infectious phages.

### Relation of Mortality Rates with Temperature

In order to investigate the variation of the mortality rate with temperature, survival curves were determined at five different temperatures ranging from 30 °C to 55 °C. Between 30 °C and 45 °C, the mortality rate *K* follows the Arrhenius equation:



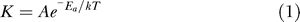



where *K* is the mortality rate, *T* is the temperature, *E _a_* is the energy of activation, *k* the Boltzman constant, and *A* is a constant specific to each reaction ( Figure 1B). For each phage, this equation gives the energy of activation of the reaction causing inactivation, i.e., the energy the system has to overcome so that the reaction occurs ( Table 1). Depending on the phage, the activation energy varies between 90 and 150 kJ/mol, which correspond to the values of reactions that result in the denaturation of globular proteins. In the case of T3, the mortality rates were determined at 55 °C, 60 °C, 62 °C, and 65 °C and proved to be much higher than expected, indicating that a different reaction is the predominant cause of inactivation. This result is in line with previously published data [ 19].

**Table 1:**
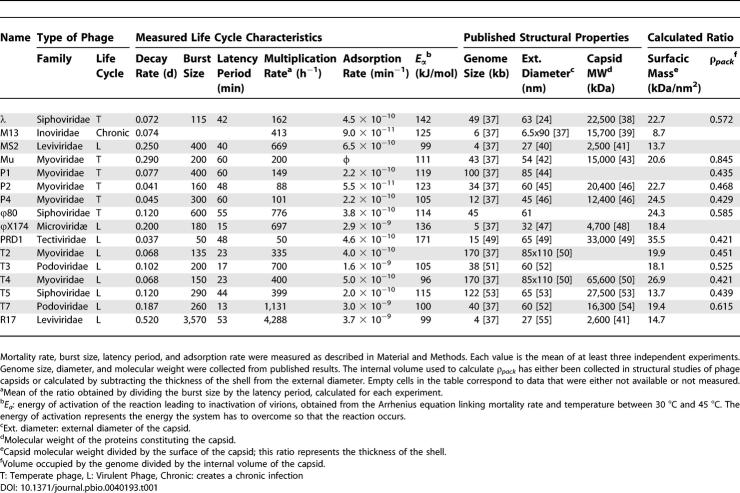
Principal Phage Parameters Tested for the Analysis of Covariance with Decay Rate

### Analysis of Covariance between Parameters Characterizing Phages

Even if the mortality of phages follows the same law for all phages, there is a great variation in the decay rate from one phage to another. To unravel what can account for this variation, for each phage, we compiled a number of structural parameters from the literature. We also calculated two associated variables that are potentially related to the stability of the capsid. The first is the density of the packaged genome, calculated only for double-stranded DNA phages because their nucleic acid shares the same chemical and physical properties. The second is the surfacic mass of the capsid, which is representative of the thickness of the proteic shell of the phage head ( Table 1). Moreover, we measured burst size, latency period, and adsorption rate for all phages, in conditions as similar as possible.

A statistical analysis of covariance revealed the existence of two groups of parameters, which can be visualized in a three-dimensional representation along axes constructed by principal components analysis, or PCA ( Figure 3). In PCA, axes are linear combinations of parameters, thus parameters that appear collinear on the figure are correlated. We can see that the size, mass, and geometry of capsids are highly correlated with genome size. This reflects the very good linear relationship between the internal volume of the capsid and the genome size over a 300-fold range (linear regression on 16 phages, *R ^2^* = 0.95, *p* < 10 ^−4^). This relation reveals important constraints about genome packaging, as the size of the capsid is determined almost entirely by genome length. Such a relation is not observed among other viruses [ 23] and thus must be caused by the specificities of phage life cycles, such as the ejection of the genome into the bacterial host at the time of infection. Indeed, phages do not enter the host cell upon infection, but instead inject only their genome into the cytoplasm.

**Figure 3:**
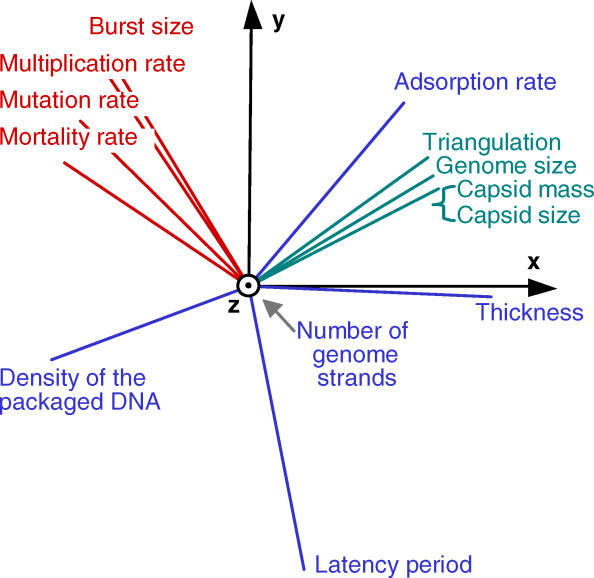
Representation of Various Phage Parameters in a PCA We can notice two groups of parameters: virion structural characteristics (green) and life history traits of phages (red). Within each group, parameters correlate highly with a nonparametric Spearman Rho test. For others correlations, see text and Figure 4.

**Figure 4:**
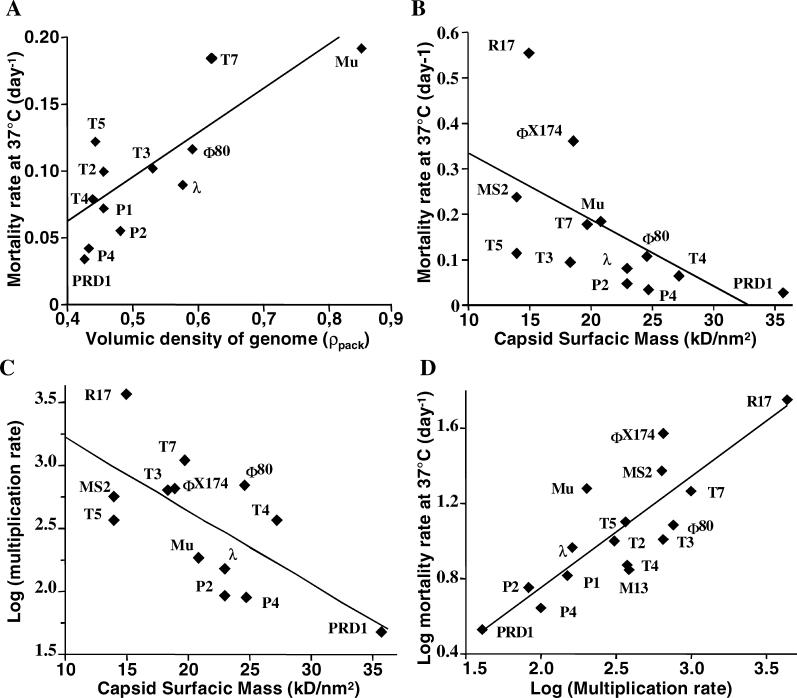
Correlations between Phage Life History Traits and Phage Particle Characteristics (A) Positive correlation between mortality rate and *ρ _pack_,* the volumic density of the packaged DNA (Linear regression, *R ^2^* = 0.67 and *p* = 0.001). *ρ _pack_* has been calculated only for phages with a double-stranded DNA genome, because the volumes of single-strand DNA and double-strand RNA are different than the volume of double-stranded DNA. *ρ _pack_* is calculated by dividing the volume of the genome by the internal volume of the capsid. (B) Negative correlation between mortality rate and the surfacic mass of the capsid, calculated by dividing the capsid molecular weight by capsid surface (linear regression, *R ^2^* = 0.35 and *p* = 0.031). Because the surfacic mass is an estimation of the thickness of the capsid, it should be related to its strength. Some phages are not represented because data are not available, or in the case of M13, because it possesses a helical geometry, and thus the constraints on the capsid are very different than for icosahedral phages. (C) Negative correlation between the multiplication rate and the surfacic mass of the capsid (linear regression, *R ^2^* = 0.46 and *p* = 0.011). (D) Positive correlation between the mortality rate and the multiplication rate. The log–log scale is for a better visualization of the results and does not modify the significance of the correlation. The line shows a linear regression characterized by *R ^2^* = 0.73 and *p* < 0.0001. Each measure was repeated at least three times independently for the determination of the multiplication and mortality rates.

As described below, the multivariate analysis revealed that the decay rate correlates with the multiplication rate of virions, but also with the density of the packaged DNA and the surfacic mass of the capsid; the latter correlates also with the multiplication rate. Other parameters are not represented in the PCA and showed no correlation, such as the energy of activation, the adsorption rate of phages on bacterial cells, or the number of viral proteins synthesized in one lytic cycle.

### Relations between Physical Constraints and Life History Traits

For phages that have a double-stranded DNA genome, the mortality rate increases significantly with the density of the packaged DNA *(ρ _pack_)* ( Figure 4A; *R ^2^* = 0.67 and *p* = 0.001 for a linear regression on 12 phages). *ρ _pack_* estimates the confinement of the genome in the capsid, by calculating the fraction of the internal volume of the capsid occupied by the genome [ 23]:



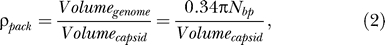



where *N _bp_* is the genome size in base pair and *Volume _capsid_* is the internal volume of the capsid in nm ^3^.

Due to the strong repulsion between charged DNA strands and the curvature of the nucleic acid chain, this confinement generates a high internal pressure. Internal pressure depends also on the presence of ions, the curvature stress of the DNA, interactions between DNA and the internal face of the capsid, the conformation state of the nucleic acid, and the spatial organization of the genome [ 23]. However, we can assume that for double-stranded DNA phages in similar environments, these parameters are roughly similar, and thus *ρ _pack_,* which varies from 0.4 to 0.7 for studied phages, should be a good approximation of the internal forces. Indeed, published DNA densities proved to be very similar for phages T7, T4, λ, P2, and P4, but smaller for PRD1 [ 24]. These measures correspond qualitatively to the ranking of values of *ρ _pack_* and confirm that this parameter is a good approximation of the genome density in the capsid. The positive correlation between the mortality rate and *ρ _pack_* suggests that a higher internal pressure is associated with a higher mortality.

Other physical parameters are involved in the stability of phage particles and, in particular, the mechanical strength of the capsid. To estimate this strength; the method used so far, and for only one phage, is the calculation of energy of the liaisons between capsomers derived from the analysis of the molecular structure [ 25]. As molecular structures are determined for only four of the phages used in this study, we approximated capsid strength by calculating the surfacic mass of the capsid shell, i.e., the molecular weight of the capsid shell divided by the surface of the capsid. In the cases in which these data were missing for a given phage, but a close relative displayed identical morphology by electron microscopy, we used the data of the related phage. Under the assumption that the density of liaisons between capsomers is somewhat constant between phages, this parameter should be related to capsid strength. Indeed, the mortality rate decreases with an increase in this parameter ( Figure 4B; *R ^2^* = 0.35 and *p* = 0.031 for a linear regression on 13 phages).

Moreover, the surfacic mass of the capsid correlates negatively with the multiplication rate ( Figure 4C; *R ^2^* = 0.46 and *p* = 0.011 for an exponential regression on 13 phages). A possible explanation is that more complex capsids may necessitate more time and resources for assembly than simpler ones. The density of the packaged DNA, however, does not correlate with the multiplication rate. This might be due to the variability in molecular processes of genome packaging between phages.

### Negative Correlation between Survival and Multiplication Rate of Phages

Despite the above-described correlations, the parameter showing the highest correlation with the mortality rate is not a structural characteristic of the capsid, but rather a major parameter of the life cycle, specifically the multiplication rate in the bacterial host ( Figure 4D; *R ^2^* = 0.73 and *p* < 0.001 for a linear regression on 16 phages). The multiplication rate was defined as the number of phage particles produced per unit of time in an infected host, calculated by dividing the burst size by the latency period. We have also tried a definition of productivity that takes into account the exponential character of phage multiplication on hosts, and leads to the same conclusions (unpublished data). An advantage of the above definition is that it is not dependent on the density of host cells. Moreover, it better reflects the rate at which cellular resources are used during virion morphogenesis.

We estimated the consequence of this positive correlation between the mortality and multiplication rates on the success of transmission of phages by calculating the persistence time *τ*, i.e., the number of days during which at least one phage particle is infectious, following one cycle of multiplication:



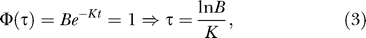



where Φ is the number of viable phage as a function of time, *K* is the decay rate, and *B* the burst size. Because of the negative correlation between the decay rate *K* and the multiplication rate, and despite an inverse relation expected from the role of the burst size *B* in the equation above, phages with the largest burst sizes are, however, the least persistent ( *p* = 0.022, Spearman Rho test). The optimal compromise between survival and multiplication might thus depend on the probability of finding a new susceptible host.

### Explanatory Power of the Correlations

The independence of the parameters that correlate with decay rate was estimated by a stepwise regression on the ten double-stranded phages for which complete structural data were available. All three parameters that have been shown to correlate individually with the decay rate—the multiplication rate, the density of the packaged DNA, and the surfacic mass of the capsid—are selected by this method and are the same in both directions of the stepwise procedure. The order of entrance of parameters is a decreasing function of their explanatory power. On these ten phages, the parameter that correlates the best with the mortality rate is the density of the packaged DNA. A model combining the three parameters accounts for 91% of the variability in the mortality rates ( Figure 5). In this multiple regression, the density of the genome and the multiplication rate contribute significantly to the coefficient of correlation, indicating that they have independent effects on inactivation. On the contrary, the remaining effect on the model of the surfacic mass is low, because this parameter correlates with the multiplication rate.

**Figure 5:**
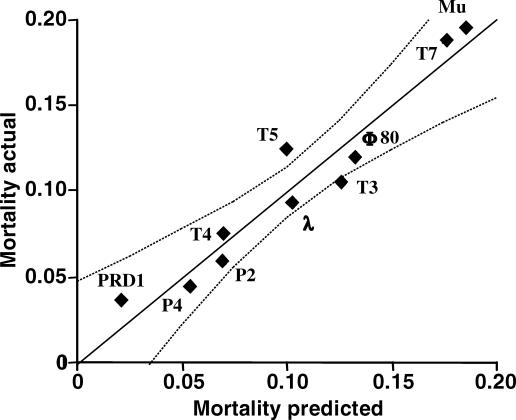
Actual Mortality Rate against Predicted Decay Rate by a Model of Multiple Regression Using the Decay Rate, *ρ _pack_,* and the Capsid Surfacic mass The estimates were identified by a stepwise regression among all parameters used. The order of entrance of parameters is an increasing function of *p*-values. The model explains 91% of the variance of the mortality rate.

Moreover, the part of the positive association between the decay and the multiplication rates that is explained by the physical properties of the virions has been estimated by looking at the correlation between the residuals of the regression between physical parameters and decay rate, and the residuals of the regression between physical parameters and multiplication rate. There is still a positive correlation between residuals ( *p* = 0.013, linear regression on ten phages), which indicates that the relation between decay and multiplication is not entirely explained by the virion physical properties that have been used in this analysis.

## Discussion

This study reveals a negative correlation between mortality and multiplication rates among phages infecting the bacteria *E. coli.* This relation parallels the trade-off between survival and reproduction revealed by life history studies of numerous species. Interestingly, this relation is observed among organisms that have diverged so much that they cannot be phylogenetically related, indicating convergent evolution. Moreover, the fact that this trade-off is present in this very simple biological situation suggests that it might be a fundamental property of evolving entities produced under constraints.

In order to maximize the data available on phages, we have selected the most well-known, representative phages of each major family of coliphages. A potential drawback is that these phages were isolated decades ago and were long maintained in various laboratories. Over this period, they probably were under selection for specific properties that might have been different for each phage. To limit this potential evolution since they were isolated, we obtained temperate phages from lysogenic strains. For lytic phages, we verified that the burst sizes and latency periods measured were almost the same as first reported. This was the case except for MS2 (400 instead of 12,000) [ 26] and PRD1 (50 instead of a few hundred) [ 27]. However, these changes did not modify the conclusions concerning the relationship between mortality and multiplication rates ( *R ^2^* = 0.56 and *p* = 0.001 with published data and *R ^2^* = 0.73 and *p* < 0.001 with measured values).

Moreover, we have measured the mortality rate of non-purified phage particles in very specific conditions: in the classical LB high-salt broth, under limited aeration and light. We can thus question whether phage life history traits in their natural environment would show the same correlation. One argument to think so is that our study give the same rank ordering among phages as found in studies of phage survival in the environment, throughout various inactivation processes [ 28– 30].

The positive correlation between mortality and multiplication rates of phages reveals an underexplored trade-off in host–parasite interactions. In most epidemiological models, transmission and virulence are assumed to be positively coupled [ 31]. Here we propose that this assumption is not necessarily valid, because transmission depends not only on the multiplication rate of the parasite, but also on survival, which is negatively correlated to the multiplication rate for coliphages.

The variation in fundamental life history traits suggests that coliphages have evolved different reproductive strategies, which would be expected to be related to their respective ecological niches. Unfortunately, if *E. coli* and its phages are the most carefully investigated genetic systems, only a few reports [ 32, 33] have yet investigated their ecology. We thus hope that this work will encourage further experiments with animal hosts of E. coli to investigate the effect of this negative correlation between multiplication and survival in vitro on the transmission of phages in more natural conditions. In particular, an interesting complement to this work would be to measure the capability of multiplication and the survival of these phages in the gut of model animals, as well as their capability of transmission between different animals.

### A Mechanistic Origin for the Trade-Off

Currently, physiology fails to fully explain the relation between survival and reproduction observed in cellular organisms [ 34]; this is probably due to the complexity of the phenomena involved. In the case of viruses, insights into the mechanistic understanding of the constraints linking inactivation and multiplication can be proposed.

Firstly, the exponential decay of phage stocks indicates that a unique kinetically dominant event leads to the inactivation of viral particles. This supports the hypothesis that for all phages, the rupture of the capsid shell is the major event leading to inactivation, and not only under stressful conditions (exposure to high temperatures or osmotic shock, for example), but also at temperatures naturally encountered by phages during their life cycles.

Moreover, the density of the packaged DNA *(ρ _pack_)* and the surfacic mass of the capsid account for 82% of the variation in the decay rate. The multiplication rate correlates with the surfacic mass of the capsid, but a part of the variability is still unaccounted for by the physical parameters considered in this study. This is probably because the multiplication rate depends on the processes involved in phage particles biosynthesis, and these processes are very different between phages [ 35, 36]. In particular, the kinetics of the assembly of the capsid, as well as the time and energy necessary to package DNA in a conformation that minimizes internal pressure, might be involved in the potential relationship between the multiplication rate and the strength of the capsid. However, the fact that the capsid surfacic mass correlates both with the decay and the multiplication rates suggests that capsid characteristics are involved into the mechanistic cause of this survival/reproduction trade-off ( Figure 6). We can hypothesize that the energy and time necessary to produce and assemble the components of phage particles might be the limiting factors in the production of virions. We hope that this report will inspire further studies analyzing the molecular mechanisms and constraints leading to trade-offs between life history traits such as reproduction and survival.

**Figure 6:**
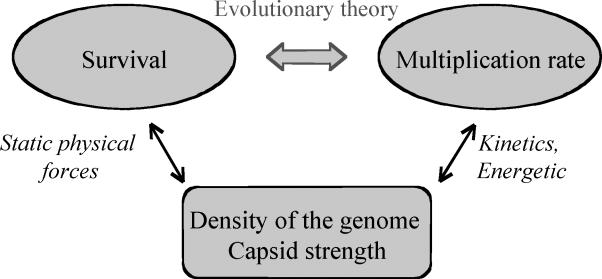
Potential Relations between Phage Survival, Multiplication Rate, and Capsid Characteristics Evolutionary theory predicts that a high decay rate is associated with an elevated multiplication rate. Forces exerted on the capsid might lead to the rupture of the head of phages, leading to their inactivation. The multiplication rate of phages is possibly associated with properties of phage particles as a consequence of kinetic and energetic considerations involved in the assembly of the capsid and/or the encapsidation of the genome.

## Materials and Methods

### Phage and bacterial strains.

We obtained the following phage and bacterial strains: phage R17 from H. W. Ackermann (Laval University); phage MS2 from J. Van Duin (Leiden University Medical Hospital); phage T5 from L. Letellier (University Paris XI); phage PRD1 from D. Bamford (University of Helsinki); phage P4 from G. Deho (University degli Studi di Milano); phages T3 and T7 from Presque Isle Cultures; phage ϕX174 from O. Tenaillon (Faculty of Medicine Xavier-Bichat); phage λ from the original E. coli K12 strain and *E. coli* C WT from the E. coli Genetic Stock Center; phages Mu and P2 from J. C. Liébart (University Paris VII); phages T2 and T4 from H. Krisch (CNRS Toulouse); phage P1 from G. Bertani; phages ϕ80 and M13 from pre-existing stocks of our laboratory; and E. coli MG1655 is the strain sequenced by Blattner.

All the above experiments were carried out in LB broth (Miller).

### Phage growth.

Phages were grown on E. coli MG1655 harboring the plasmid F, except for experiments with the following phages: T7 which is inhibited by the plamid F, had been grown on *E coli* MG1655; ϕX174 which uses *E. coli* C LPS for adsorption had been grown on E. coli C WT; PRD1 which uses plasmid of group P, N, or W as receptor was grown on *E coli* MG1655 RP4; and P4, which is a satellite of P2, was grown on E. coli MG1655 F lysogenic for P2. For the production of phage suspensions, 10 ml of mid–exponential phase E. coli cultures are infected with phage and incubated with shaking at 37 °C. When the lysis is visible, the culture is centrifuged and the supernatant is passed through a 0.22-μm pore filter to eliminate bacterial cells.

### Phage inactivation.

Inactivation, which is defined as the loss of ability to form a plaque of lysis on a lawn of susceptible hosts, was studied by incubating samples of a phage lysate diluted in LB in 1.5-ml tubes at 30 °C, 37 °C, 42 °C, 45 °C, and 55 °C. Over periods of weeks, the number of viable phage was repeatedly assessed by measuring plaque forming units (PFU), each measurement coming from an individual sample. Mortality rate was calculated by fitting an exponential curve to the decay of the phages.

### Phage adsorption.

The phage adsorption constant was determined by mixing a mid–log phase E. coli (OD _600_ between 0.2 and 0.3) susceptible culture and the appropriate number of phage to ensure a multiplicity of infection (m. o. i.) of 10 ^−4^. The mix tubes were incubated at 37 °C, and then every 2–5 min, a tube was centrifuged for 2 min at maximum bench centrifuge speed. The number of free phage in the supernatant was estimated by plaque assay. The phage adsorption constant was estimated by fitting an exponential curve to the evolution of the free-phage population with time, with the adsorption rate being defined as the value of the exponential fit divided by the number of colony-forming units at the time of mixing, as determined by plaque assay.

### Burst size and latency period.

Burst size was determined by incubating 10 ml of a mid–exponential phase E. coli culture on ice for 10 min, centrifuging, and then resuspending the bacterial culture in minimal medium (M9) and mixing with phage (multiplicity of infection = 0.01) for 7 min at 37 °C. The mix was then diluted 100-fold and 10,000-fold in LB at 37 °C. The number of free phage was evaluated by plaque assay after centrifugation. Samples of the dilutions mix were taken repeatedly throughout time and assayed immediately for plaque-forming units. The burst size is the factor between final phage number and initial phage number, subtracting the number of non-adsorbed phage at time of dilution. Latency period is the time at which 50% of the virions have been released.

### Electron microscopy.

Aliquots (10 μl) of phage suspension were adsorbed onto 400-mesh carbon-coated copper grids, washed on a 10-μl drop of phage buffer (pH 7.1), and negatively stained in 1.5% (wt/vol) uranyl acetate. Samples were observed in a CM120 transmission electron microscope (Philips, Eindhoven, The Netherlands) equipped with a minimum electron dosage system. Low-dose micrographs were recorded on a 1k × 1k CCD Multiscan Gatan camera (Gatan, Pleasanton, California, United States) at an accelerating electron voltage of 120 kV, and at nominal magnifications from 6,000× to 17,000×. Average defocus values were −1,200 nm.

### Phage parameters and statistical analysis.

We made a systematic analysis of correlations between the decay rate and the parameters describing phages characteristics presented in Table 1. For ease in comparing the different phages, we defined the multiplication rate as the burst size divided by the latency period. This measure provides a growth rate that is not scaled to generation time, which varies between phages. Phage capsids are considered spherical for the calculation of the volume, expect for phages T4 and T2, elongated phages, which are considered to be a capped cylinder, and M13, a filamentous virus, which is considered to be an elongated cylinder. Depending on the available data, values of the molecular weight of capsids had been obtained by direct mass measurements of capsids, or by the addition of the molecular weight of every protein constituting the capsid shell.

The statistical analysis was made with JMP IN 4 (SAS Institute, Cary, North Carolina, United States). Regressions are estimated by least squares methods. They were systematically performed on all the phages for which the relevant data were available.

## Abbreviations

ρ _*pack*_: density of the packaged DNA
PCA: principal components analysis
