# Correction: Viruses' Life History: Towards a Mechanistic Basis of a Trade-Off between Survival and Reproduction among Phages

**DOI:** 10.1371/journal.pbio.0040273

**Published:** 2006-08-15

**Authors:** François Taddei, Marianne De Paepe

## Abstract

A comparison of life-history traits of 16 phages infecting *E. coli* reveals that although these viruses don't age, there is a trade-off between mortality and growth rate, which parallels that observed in many other species.

In *PLoS Biology*, volume 4, issue 7: DOI: 10.1371/journal.pbio.0040193

In the abstract, the mortality rate is described as being negatively correlated with multiplication rate, whereas it is positively correlated.

